# Supplementary material for: Symmetries and cluster synchronization in multilayer networks
Source: Nat Commun. 2020 Jun 23;11:3179. doi: 10.1038/s41467-020-16343-0 (PMC7311444; doi:10.1038/s41467-020-16343-0)
Supplement: Supplementary file 1 — Supplementary Information [file 41467_2020_16343_MOESM1_ESM.pdf]

Supplementary Information for
Symmetries and Cluster Synchronization in Multilayer Networks

Della Rossa *et al.*

Supplementary Note 1: Datasets

In Table 1 of the main manuscript we compute the symmetries of several real multilayer networks. In Supplementary Table 1 we provide an extensive description of each dataset. In particular, we list information about what the nodes, the edges, and the layers represent in each dataset, as well as whether the edges are weighted or directed.

To provide a better connection between real multilayer networks and cluster synchronization, we focus on the US power-grid dataset [1]. The network is composed of $N^b = 4492$ buses (layer b) and $N^g = 449$ generators (layer g), which are connected in the power-grid network via both interlayer end intralayer links. We assume that all the buses have identical power demand $P_i^b = -100\ kW$, while each generator can supply roughly $P_i^g = 1MW$. We thus suppose to have $N^b + N^g$ rotatory machines, with equal inertia ($I_i^b = I_i^g = 10^7 ton\ m^{-2}$) and damping coefficient ($\gamma_i^b = \gamma_i^g = 10^5 ton\ s\ m^{-2}$). Finally, we assume the admittance on all the power-grid connections is equal to $10^4 MS$. The power-flow in the network can be described by the well-known swing equation [2, 3, 4, 5], and, the system dynamics is described by

$$\begin{aligned} \dot{x}_{i,1}^{\alpha} &= x_{i,2} \\ I_i^{\alpha}\dot{x}_{i,2}^{\alpha} &= P_i^{\alpha} - \gamma_i^{\alpha} x_{i,2}^{\alpha} + \sum_{j=1}^{N^{\alpha}} A_{ij}^{\alpha\alpha} \sin(x_j^{\alpha} - x_i^{\alpha}) + \sum_{j=1}^{N^{\beta}} A_{ij}^{\alpha\beta} \sin(x_j^{\beta} - x_i^{\alpha}) \end{aligned}$$

where $x_{i,1}^{\alpha}$, $i = 1, \ldots, N^{\alpha}$, $\alpha = b, g$ ($\beta = g, b$) is the phase of the signal at the bus/generator with respect to a nominal signal. Figure 1 shows the power-grid network (left) together with the time evolutions of the phases for the four largest clusters of the multi-layer network (right). As can be seen from the right panel, after a transient, the nodes in each cluster reach CS.

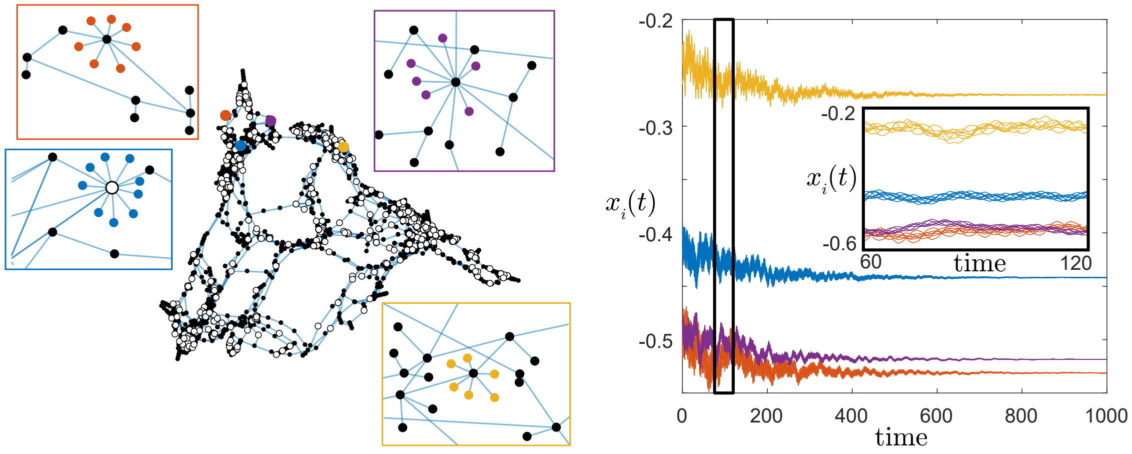

Supplementary Figure 1: (left) The US power grid network from [1]. Filled nodes are loads, while empty nodes are generators. Nodes from the four largest clusters of the network are colored the same, either yellow, or blue, or purple, or red. Each inset is a magnification of the image to better show each one of these clusters. (right) Time evolution of the phases of the loads in each cluster.

Name	Type	Node Info	Edge Info	Layer Info
London [6]	Transportation	Each node represents a train station in London.	• Edges encode existing routes between stations.<br>• Edges weights indicate multiple routes.<br>• Edges are undirected	3 layers:<br>1. Underground aggregated lines<br>2. Overground 3. DLR
EU-Air [7]	Transportation	Each node represents an airport.	• Edges stand for direct flights between two airports<br>• Edges are unweighted (either flight is present or not)<br>• Edges are undirected.	37 layers<br>• Each layer corresponds to a different airline.
ARXIV-Netscience [8]	Coauthorship	Each node represents a co-author	• Edges stand for coauthorship connections<br>• Edges are weighted.<br>• Edges are undirected.	13 layers<br>• Each layer corresponds to a different arXiv category.
PIERRE AUGER [8]	Coauthorship	Each node represents a co-author	• Edges stand for coauthorship connections<br>• Edges are weighted.<br>• Edges are undirected.	16 layers<br>• Each layer corresponds to a different working task within the Pierre Auger collaboration.
CKM Physicians [9]	Social	Each node represents a physician.	• Edges represent information being exchanged regarding a specific drug.<br>• Edges are unweighted.<br>• Edges are directed.	3 layers<br>• Each layer corresponds to a different type of social interaction:<br>1. Advice 2. Discussion 3. Friend
BOS Linnaeus [10, 11]	Genetic	Each node represents a protein.	• Edges represent protein-protein interactions.<br>• Edges are unweighted.<br>• Edges are directed.	3 layers<br>• Each layer corresponds to interactions of a different nature.
CANDIDA [10, 11]	Genetic	Each node represents a protein.	• Edges represent protein-protein interactions.<br>• Edges are unweighted.<br>• Edges are directed.	7 layers<br>• Each layer corresponds to interactions of a different nature.
DANIORERIO [10, 11]	Genetic	Each node represents a protein.	• Edges represent protein-protein interactions.<br>• Edges are unweighted.<br>• Edges are directed.	5 layers<br>• Each layer corresponds to interactions of a different nature.
US Power-grid [1]	Power-grid	Each node represents either a generator or a load.	• Edges represent line connections.<br>• Edges are unweighted.<br>• Edges are undirected.	2 layers<br>• Each layer corresponds to a different node kind (generator or load.)

Supplementary Table 1: Detailed description of the real multilayer networks considered in Table 1 of the main manuscript.

Supplementary Note 2: Symmetries of Model Multiplex Networks

In this section, we compute the symmetries of model multiplex networks, with $M = 2$ layers, constructed as follows. Both layers are Scale Free (SF) networks generated by using the static model [12] as implemented in [13], with the same average degree $\kappa_{av} = 5$ and the same number of nodes $\mathcal{N} = 1000$, but variable power-law degree distribution exponents γ_1 and γ_2. We randomly matched each node $i = 1, ..., \mathcal{N}$ from layer α with another node from layer β to obtain a multiplex network. The inter-layer coupling $A_{ij}^{\alpha\beta} = A_{ji}^{\alpha\beta} = 1$ if node i from layer α was matched to node j from layer β, and $A_{ij}^{\alpha\beta} = A_{ji}^{\alpha\beta} = 0$ otherwise. In case the resulting multiplex network was not connected, only the largest connected component was considered.

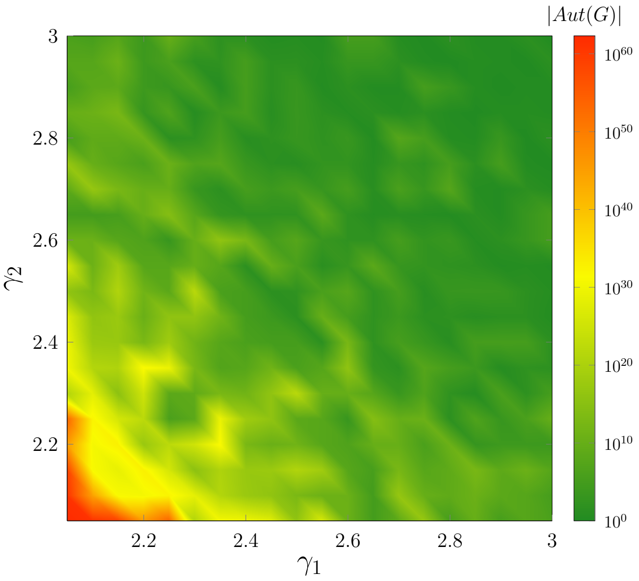

Supplementary Figure 2: Number of symmetries of multiplex model networks. Multiplex model networks were constructed with $M = 2$ layers, by randomly matching nodes from two layers, where both layers are scale-free networks with the same average degree $\kappa_{\mathrm{avg}} = 5$ and with the same number of nodes $N = 1000$, but with variable degree distribution exponents γ_1 and γ_2. For each pair of exponents (γ_1, γ_2) the number of symmetries plotted is an average over 200 realizations.

Supplementary Figure 2 shows the number of symmetries of the resulting multiplex network as a function of the pair (γ_1, γ_2), $2 \le \gamma_1, \gamma_2 \le 3$, averaged over 200 realizations. We observe a large number of symmetries when both the power low exponents γ_1 and γ_2 are small. When both exponents exceed 2.6, the number of symmetries (except for the identity) is typically zero. The case of Erdos Renyi layers is recovered in the limit of very large exponent γ, for which symmetries are very rare.

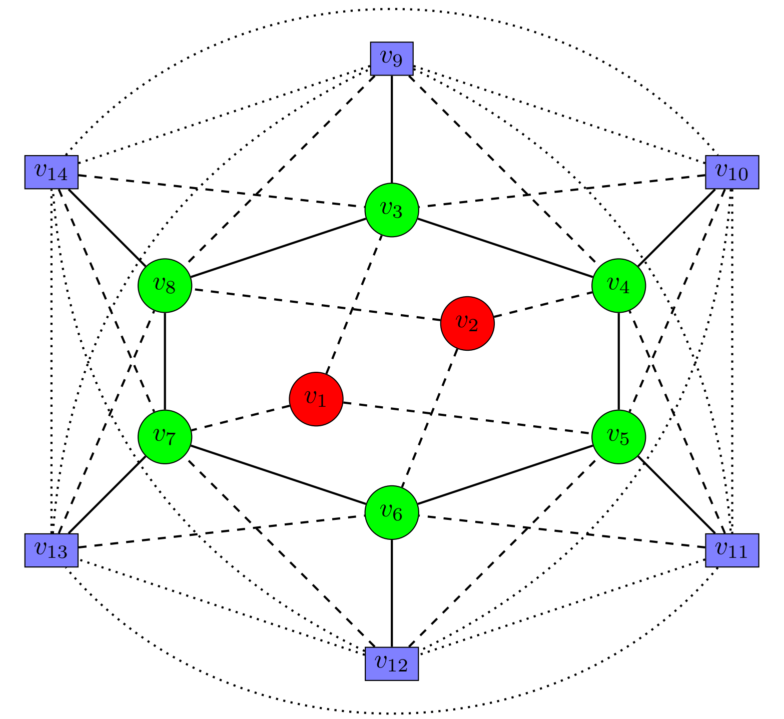

Supplementary Figure 3: An example of a multi-layer graph, color coded according to the orbits of its automorphism group. There are two types of nodes, denoted as circles and rectangles. There are also three types of edges, denoted as solid, dashed, and dotted lines connecting pairs of nodes. The orbits of the automorphism group are colored as red, green, and blue.

Supplementary Note 3: Generating Multilayer Networks with Symmetries

Consider a multilayer network with M node colors (or types) and K edge colors (or types), where, if the nodes or edges are not colored, then $M = 1$ or $K = 1$, respectively. Let the set of nodes of type j be denoted X^α, $\alpha = 1, \ldots, M$, and the set of edges of type k be denoted $\mathcal{E}^k$, $k = 1, \ldots, K$. Let $Aut(\mathcal{G})$ be the automorphism group of the graph as described in the main text and let $\mathcal{O}$ be the orbital partition of the nodes induced by the automorphism group. The orbital partition consists of $|\mathcal{O}| = n_o$ orbits, and each orbit $\mathcal{O}_k$ consists of nodes of the same type only.

An example of the structures defined is shown in Supplementary Figure 3. The graph consists of a total of 14 nodes and a total of 42 edges, with $M = 2$ node types (circles and rectangles) and $K = 3$ edge types (solid, dashed, and dotted). The nodes are colored according to their orbits in the automorphism group; red, green, and purple. The orbits are found using `nauty`.

The orbital partition of a graph can be represented by a *quotient graph*, that is, a multilayer

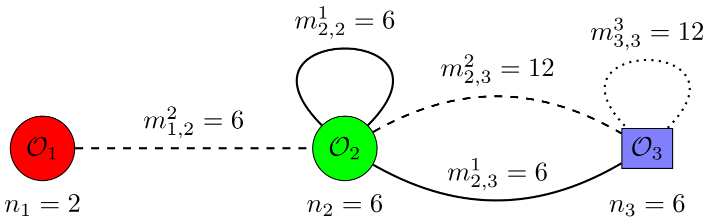

Supplementary Figure 4: The quotient graph of the graph shown in Supplementary Figure 3. The orbits are colored according to their representative population, and they have the shape of their constituent nodes as well. The quotient edges are drawn according to their representative type as well, and the number of edges represented by each quotient graph are shown as well.

graph $\mathcal{Q} = (\mathcal{O}, \mathcal{F})$ where the nodes are the orbits of the original graph (colored according to the single color of its constituents) and the edges are,

$$\mathcal{F}^{\ell}_{j,k} = \{(v_a, v_b) \in \mathcal{E}_\ell | v_a \in \mathcal{O}_j \wedge v_b \in \mathcal{O}_k\} \tag{1}$$

The minimal representation of the original graph only preserves the size of these sets, that is, $n_k = |\mathcal{O}_k|$ and $m^{\ell}_{j,k} = |\mathcal{F}^{\ell}_{j,k}|$, losing the detailed information of the original set of edges.

The quotient graph of the multilayer graph shown in Supplementary Figure 3 is shown in Supplementary Figure 4. The quotient nodes, labeled $\mathcal{O}_1$, $\mathcal{O}_2$, and $\mathcal{O}_3$, have the shape of their constituent nodes and are colored according to the orbit that they represent. The original population of each orbit is shown below each node, and the number of edges of each type passing between nodes in each orbit is shown above each edge.

Going from a graph and its automorphism group to the representative quotient graph is straightforward computationally. The reverse process is not straightforward, and a single quotient graph represents an infinite number of original graphs. Before proceeding though, the definition of a quotient graph must be made more restrictive, that is, the set of orbit populations n_k, $k = 1, \ldots, n_o$, and the number of intra- and inter-orbit edges $m^{\ell}_{j,k}$, $j, k = 1, \ldots, n_o$, $\ell = 1, \ldots, K$, must satisfy a set of conditions such that it is capable of representing some original graph.

The requirements can be succinctly stated as the following four conditions.

- The number of inter-cluster edges, $m^{\ell}_{j,k}$, $j \neq k$, for each edge color ℓ, must be divisible by both n_j and n_k. Note that if $m^{\ell}_{j,k} = 0$ this is trivially satisfied.
- The number of inter-cluster edges must be less than or equal to the maximum number of inter-cluster edges, that is, $m^{\ell}_{j,k} \leq n_j n_k$, $\ell = 1, \ldots, K$, $j \neq k$.
- Twice the number of intra-cluster edges, $2m^{\ell}_{j,j}$ must be divisible by the number of nodes n_j, for $j = 1, \ldots, n_j$ and $\ell = 1, \ldots, K$.
- The number of intra-cluster edges must be less than the maximum possible number of edges, that is, $m^{\ell}_{j,j} \leq \frac{n_j(n_j-1)}{2}$.

These conditions can be expressed mathematically as,

$$\begin{cases} m^{\ell}_{j,k} = z^{\ell}_{j,k}\text{LCM}(n_j, n_k), & j \neq k \\ 0 \leq m^{\ell}_{j,k} \leq n_j n_k, & j \neq k \\ z^{\ell}_{j,k} \geq 0 & j \neq k \\ 2m^{\ell}_{j,j} = z^{\ell}_{j,j} n_j & \\ 0 \leq m^{\ell}_{j,j} \leq \dfrac{n_j(n_j-1)}{2} & \\ z_{j,j} \geq 0, & \end{cases} \tag{2}$$

where in the above equation $\text{LCM}(n_j, n_k)$ stands for the *least common multiple* of the integers n_j and n_k. If these four conditions are met, then the quotient graph is capable of representing at least one original graph such that the quotient graph represents the orbits of its automorphism group.

The question remains how to construct the desired graph. To do this, we start by creating n_o sets of disconnected nodes $\mathcal{O}_k$, $k = 1, \ldots, n_o$, such that $\mathcal{V} = \bigcup_{k=1}^{n_o} \mathcal{O}_k$ colored according to the colors of the orbits. For ease of notation in the following descriptions, we label the nodes according to their originating orbit, v^j_i, $i = 1, \ldots, n_j$, $j = 1, \ldots, n_o$. The edges are added so that the resulting graph's automorphism group contains permutations of the type,

$$\exists \pi \in Aut(\mathcal{G}) \quad \text{such that} \quad \pi(v^j_i) = v^j_{(i+q) \bmod n_j}, \quad i = 1, \ldots, n_j, \quad j = 1, \ldots, n_o, \quad \forall q \in \mathbb{Z} \tag{3}$$

which can be thought of as cycles of order q within each orbit. We then add the intra-orbit edges for each value $m^{\ell}_{j,j} > 0$, $j = 1, \ldots, n_o$ and $\ell = 1, \ldots, K$. To do this, let $k^{\ell}_{j,j} = \frac{2m^{\ell}_{j,j}}{n_j}$ be the intra-cluster degree of color ℓ of orbit j.

- If $k^{\ell}_{j,j}$ is odd, then we choose $\frac{k^{\ell}_{j,j}-1}{2}$ unique integers between 1 and $\frac{n_j}{2}$, labeled c_i, $i = 1, \ldots, \frac{k^{\ell}_{j,j}-1}{2}$. The set of neighbors for a node in this orbit with edges of type ℓ are,

$$\forall v_a \in \mathcal{O}_j \quad \mathcal{N}^{\ell}_{a,j} = \left\{ v^j_{a \pm c_i \bmod n_j} | \forall c_i \right\} \cup \left\{ v^j_{a+n_j \bmod n_j} \right\} \tag{4}$$

- If $k^{\ell}_{j,j}$ is even, then we choose $\frac{k^{\ell}_{j,j}}{2}$ unique integers between 1 and $\lfloor \frac{n_j}{2} \rfloor$, labeled c_i, $i = 1, \ldots, \frac{k^{\ell}_{j,j}}{2}$. The set of neighbors for a node in this orbit with edges of type ℓ are,

$$\forall v_a \in \mathcal{O}_j \quad \mathcal{N}^{\ell}_{a,j} = \left\{ v^j_{a \pm c_i \bmod n_j} | \forall c_i \right\} \tag{5}$$

Note that any cycle of order q of the nodes in the orbits does not affect the set of edges.

We now turn to the inter-cluster edges, which is somewhat more involved. Let $m^{\ell}_{j,k}$ be the number of edges between orbits $\mathcal{O}_j$ and $\mathcal{O}_k$ of color ℓ and let there be $n_j = |\mathcal{O}_j|$ nodes in orbit j and $n_k = |\mathcal{O}_k|$ nodes in orbit k. Let $g = \text{GCD}(n_j, n_k)$ be the *greatest common divisor* of integers n_j and n_k so that there exist two other integers, d_j and d_k, such that,

$$n_j = d_j g \quad \text{and} \quad n_k = d_k g \tag{6}$$

Define $h = \frac{m^{\ell}_{j,k} g}{n_j n_k}$ and a sequence of integers $f = \{f_1, \dots, f_h\}$ such that,

$$\sum_{i=1}^{h} f_i = g \tag{7}$$

If $v^j_a \in \mathcal{O}_j$, then its neighbors in $\mathcal{O}_k$ can be listed as v^k_b where

$$b = \left(a + r_1 g + \sum_{i=1}^{r_2} f_i \right) \bmod n_k, \quad r_1 = 0, \dots, d_k - 1, \quad r_2 = 1, \dots, h \tag{8}$$

or, vice versa, if $v_b \in \mathcal{O}_k$ then its neighbors in orbit $v_a \in \mathcal{O}_j$ with edges of color ℓ are,

$$a = \left(b + r_3 g + \sum_{i=1}^{r_4} f_{h-i+1} \right) \bmod n_j, \quad r_3 = 0, \dots, d_j - 1, \quad r_4 = 1, \dots, h \tag{9}$$

Proofs of these expressions for the set of edges can be found in [14, 15]

A single quotient graph may represent multiple original graphs. Different choices of the values c_i for the intra-orbit edges and the values f_i in the inter-orbit edges can lead to a sampling of the possible graphs. Also, if we determine a set of populations n_j, $j = 1, \dots, n_o$, and edge amounts $m^{\ell}_{j,k}$ for each edge between quotient nodes, the quotient graph can be scaled up by multiplying every quantity by a positive integer. It is trivial to show that all of the constraints hold when multiplying the quantities by a constant positive integer.

Supplementary Note 4: Effects on CS Stability of Heterogeneous Systems in Different Layers

The equations describing the dynamics of a multilayer network are,

$$\dot{\mathbf{x}}^{\alpha}_i = \mathbf{F}^{\alpha}(\mathbf{x}^{\alpha}_i) + \sigma^{\alpha\alpha} \sum_{j=1}^{N^{\alpha}} A^{\alpha\alpha}_{ij} \mathbf{H}^{\alpha\alpha}(\mathbf{x}^{\alpha}_j) + \sum_{\beta \neq \alpha} \sigma^{\alpha\beta} \sum_{j=1}^{N^{\beta}} A^{\alpha\beta}_{ij} \mathbf{H}^{\alpha\beta}(\mathbf{x}^{\beta}_j) \tag{10}$$

For simplicity we focus on the case of $M = 2$ layers, and set the matrices $A^{\alpha\alpha}, A^{\beta\beta}, A^{\alpha\beta} = (A^{\beta\alpha})^T$ to represent the multilayer network in Figure 1 of the main manuscript. An example with the Van der Pol oscillators is presented in Figure 2 of the main manuscript.

Chaotic systems

Here we consider the cases of the Lorenz and of the Roessler oscillators. For all the cases considered, we see that increasing the level of heterogeneity between the oscillators in different layers leads to loss of stability of DLS blocks. In this section we further investigate the effects of varying heterogeneity between the systems in the two layers on stability of DLS's. In the case of the Lorenz oscillator, we set $n^{\alpha} = n^{\beta} = 3$, $\mathbf{x}^{\alpha} = [x^{\alpha}, y^{\alpha}, z^{\alpha}]$, $\mathbf{x}^{\beta} = [x^{\beta}, y^{\beta}, z^{\beta}]$,

$$\mathbf{F}^{\alpha}(\mathbf{x}^{\alpha}) = \begin{bmatrix} c^{\alpha}(y^{\alpha} - x^{\alpha}) \\ -x^{\alpha} z^{\alpha} + 28 x^{\alpha} - y^{\alpha} \\ x^{\alpha} y^{\alpha} - 8/3 z^{\alpha} \end{bmatrix}, \qquad \mathbf{F}^{\beta}(\mathbf{x}^{\beta}) = \begin{bmatrix} c^{\beta}(y^{\beta} - x^{\beta}) \\ -x^{\beta} z^{\beta} + 28 x^{\beta} - y^{\beta} \\ x^{\beta} y^{\beta} - 8/3 z^{\beta} \end{bmatrix},$$

which both correspond to the dynamics of the Lorenz oscillator, with layer-dependent parameters c^α and c^β. Moreover, we set

$$\mathbf{H}^{\alpha\beta}(\mathbf{x}) = \begin{pmatrix} 1 & 0 & 0 \\ 0 & 1 & 0 \\ 0 & 0 & 0 \end{pmatrix} \mathbf{x},$$

for all pairs $\alpha, \beta = 1, 2$, $\sigma^{\alpha\beta} = -2$ for all pairs $\alpha, \beta = 1, 2$. We set the layer-dependent parameters $c^\alpha = 10(1 + \delta_c)$ and $c^\beta = 10(1 - \delta_c)$, so that $\delta_c = 0$ corresponds to identical systems in the two layers and increasing values of δ_c indicate the systems in the two layers are increasingly different. We then numerically investigate stability of DLS symmetries as the parameter δ_c is increased. This can be seen in panel A) of Supplementary Figure 5, which shows that synchronization between oscillators 1 and 3 from the α layer and synchronization between oscillators 1 and 3 from the β layer is simultaneously lost for $\delta_c \gtrapprox 0.25$.

We then consider the case of the Roessler oscillators, for which $n^\alpha = n^\beta = 3$, $\mathbf{x}^\alpha = [x^\alpha, y^\alpha, z^\alpha]$, $\mathbf{x}^\beta = [x^\beta, y^\beta, z^\beta]$,

$$\mathbf{F}^\alpha(\mathbf{x}^\alpha) = \begin{bmatrix} -y^\alpha - z^\alpha \\ x^\alpha + 0.2c^\alpha y^\alpha \\ 0.2 + (x^\alpha - 3)z^\alpha \end{bmatrix}, \qquad \mathbf{F}^\beta(\mathbf{x}^\beta) = \begin{bmatrix} -y^\beta - z^\beta \\ x^\beta + 0.2c^\beta y^\beta \\ 0.2 + (x^\beta - 3)z^\beta \end{bmatrix}.$$

Moreover, we set

$$\mathbf{H}^{\alpha\beta}(\mathbf{x}) = \begin{pmatrix} 0 & 0 & 0 \\ 0 & 1 & 0 \\ 0 & 0 & 0 \end{pmatrix} \mathbf{x},$$

for all pairs $\alpha, \beta = 1, 2$, $\sigma^{\alpha\beta} = -0.04$ for all pairs $\alpha, \beta = 1, 2$. We set the layer-dependent parameters $c^\alpha = 10(1+\delta_c)$ and $c^\beta = 10(1-\delta_c)$, so that $\delta_c = 0$ corresponds to identical systems in the two layers and increasing values of δ_c indicate the systems in the two layers are increasingly different. We then numerically investigate stability of DLS symmetries as the parameter δ_c is increased. Panel B) of Supplementary Figure 5 shows that synchronization between oscillators 1 and 3 from the α layer and synchronization between oscillators 1 and 3 from the β layer is simultaneously lost for $\delta_c \gtrapprox 0.06$.

Time-varying heterogeneity

We now reconsider the example with the Van der Pol oscillator illustrated in Figure 2 of the main manuscript. We copy below the functions **F** and **H** for the reader's convenience:

$$\mathbf{F}^\alpha(\mathbf{x}^\alpha) = \begin{bmatrix} y^\alpha \\ -x^\alpha - 0.2y^\alpha[(x^\alpha)^2 - c^\alpha] \end{bmatrix}, \quad \mathbf{F}^\beta(\mathbf{x}^\beta) = \begin{bmatrix} y^\beta \\ -x^\beta - 0.2y^\beta[(x^\beta)^2 - c^\beta] \end{bmatrix},$$

$$\mathbf{H}^{\alpha\beta}(\mathbf{x}) = \begin{pmatrix} -1 & 0 \\ 0 & 0 \end{pmatrix} \mathbf{x},$$

and we set, for all pairs $\alpha, \beta = 1, 2$, $\sigma^{\alpha\beta} = 0.15$. We set the layer-dependent parameters $c^\alpha = (1+\delta_c)$ and $c^\beta = (1-\delta_c)(1+\varepsilon \sin \omega t)$, with $\delta_c = 0.35$. In this way, when $\varepsilon = 0$ we are in the situation already considered in the main text, namely Figure 2 of the main manuscript shows that for $\delta_c = 0.35$ (and $\varepsilon = 0$) cluster synchronization is achieved in both layers. We now consider how the emergence of cluster synchronization is affected if the parameter variation is periodic in time, for different intensity of the variation ε and frequency ω. Notice that for increasing ε the time-averaged parametric difference between the systems in the two layers remains unvaried.

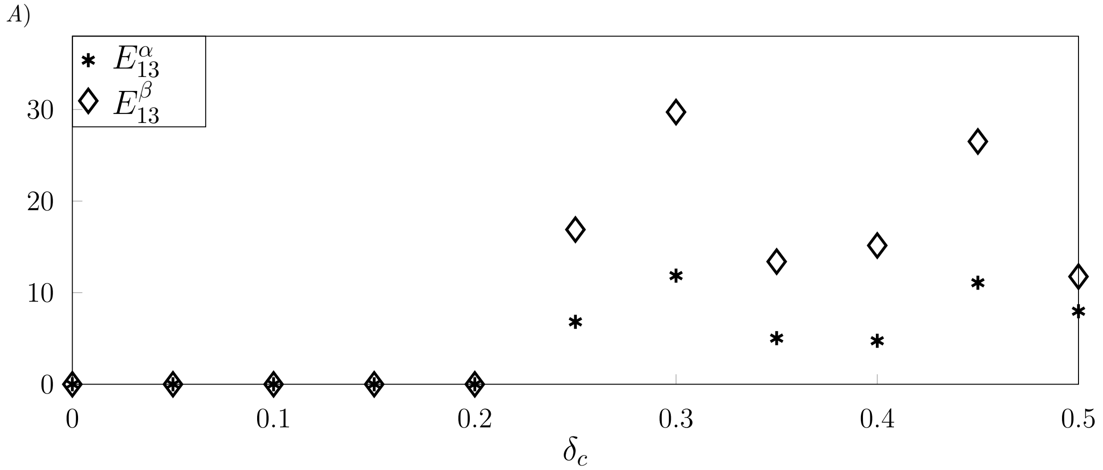

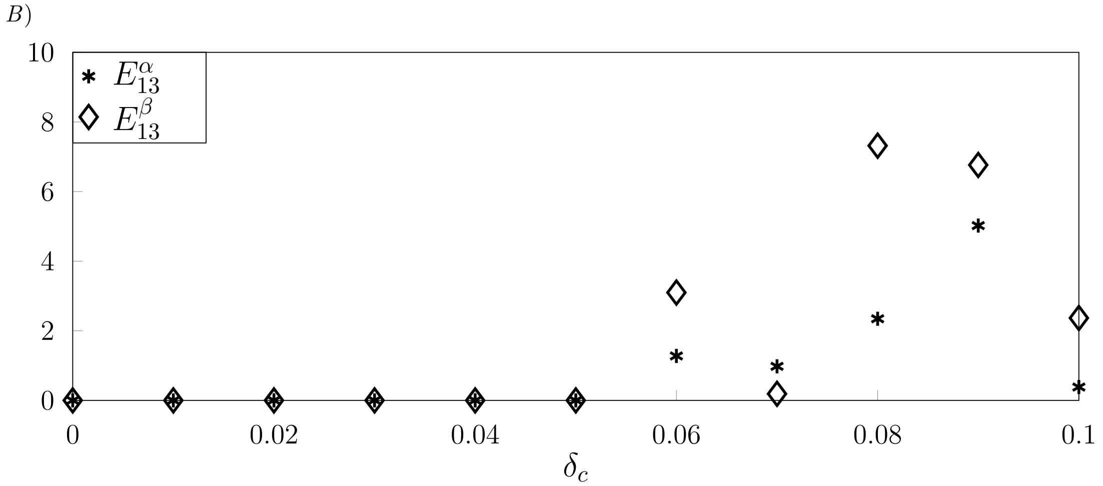

Supplementary Figure 5: A) Lorenz oscillators: synchronization errors E_{13}^{α} (asterisks) and E_{13}^{β} (diamonds) vs the parameter δ_c. $\delta_c = 0$ corresponds to identical systems between the α and in the β layers. Increasing values of δ_c are for increasingly different systems in the two layers. Synchronization is lost in both layers for $\delta_c \gtrapprox 0.25$. B) Roessler oscillators. In this case synchronization is lost in both layers for $\delta_c \gtrapprox 0.06$.

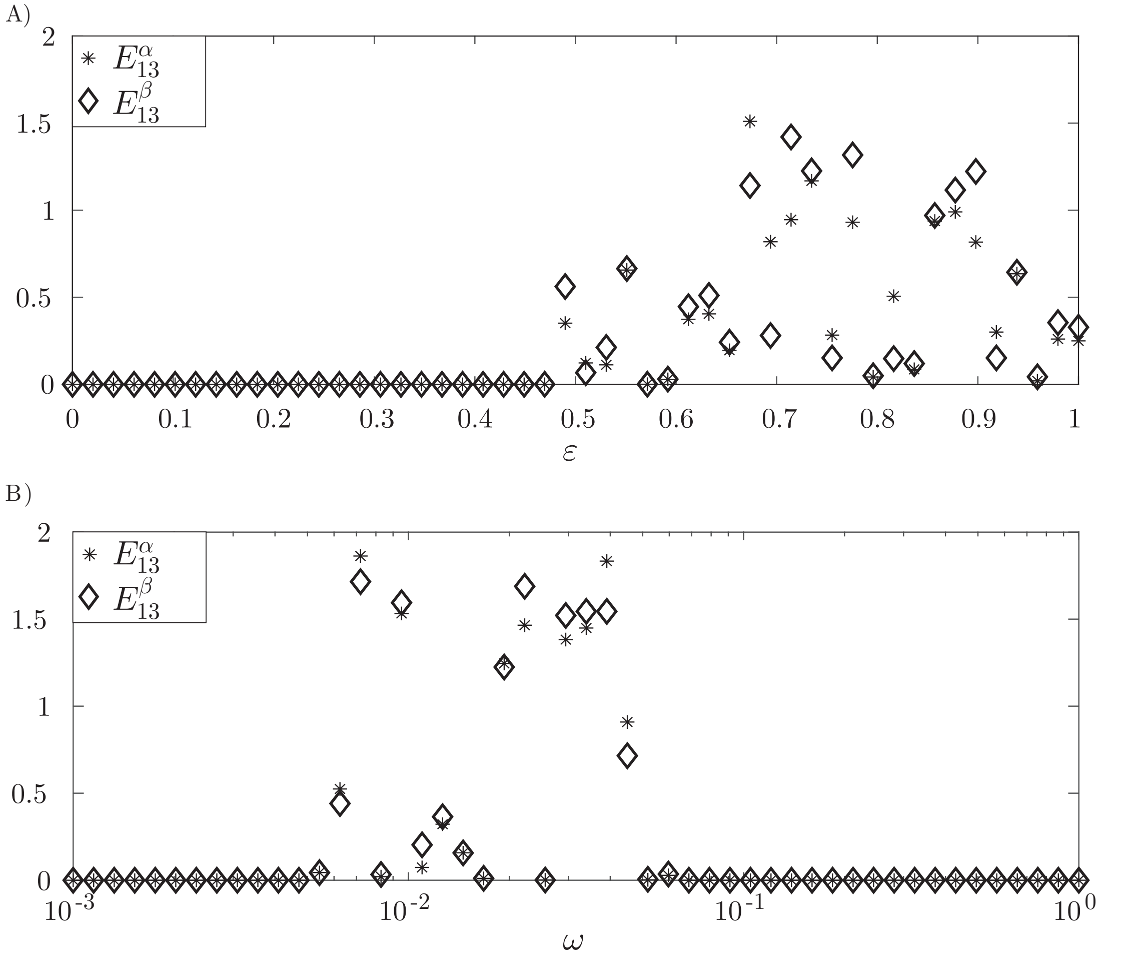

Supplementary Figure 6: Synchronization errors E_{13}^{α} (asterisks) and E_{13}^{β} (diamonds) vs A) the amplitude of a time varying difference ε with $\omega = 0.04$ and B) the frequency ω of the time varying parameter with $\varepsilon = 0.7$.

Supplementary Figure 6 shows the results of our numerical experiments. In panel A) we plot the cluster synchronization error for increasing time-varying amplitude ε when $\omega = 0.04$. We see that cluster-synchronization is achieved as long as $\varepsilon \lessapprox 0.5$, and thus it is robust with respect to small periodic time-varying heterogeneity, while it is lost for larger values of ε. Panel B) instead plots the cluster synchronization error for different frequencies ω at a fixed amplitude $\varepsilon = 0.7$. The case with $\omega = 0$ corresponds to a case without time-varying parameters. Cluster-synchronization is achieved when the time-varying perturbation is either sufficiently slow or fast and it is lost when the time-varying perturbation has a frequency that is comparable with the frequencies of the two Van der Pol systems, which is about 0.08.

Effects of noise on cluster synchronization

Here we study the effects of noise on cluster synchronization, e.g. due to some external unpredictable factor that affects a layer parameter. To do that, we consider again that the β-layer parameter is not constant but is affected by noise $c^\beta(t) = (1 - \delta_c)(1 + \varepsilon\zeta(t))$, where $\zeta(t)$ is a noisy term with a prescribed spectrum and standard deviation equal to 1. We consider three kinds of noise: a standard white noise (a signal with equal power in every band of a given bandwidth–power spectral density), a pink noise (a signal with equal power in bands that are proportionally wide, i.e. with a spectral power density that decreases by 3 dB per octave), and a blue noise (a signal with spectral power density that increases by 3 dB per octave).

Supplementary Figure 7 shows the results of our numerical experiments. In panel A) $\zeta(t)$ is a white noise. On the left panel, we show its power spectral density, while on the right panel we plot the DLS synchronization error against the noise intensity ε. Panels B) and panels C) show the same information, but for the cases of a pink and a blue noise, respectively. We see in all the cases that cluster synchronization is achieved if the noise intensity is sufficiently small, but is lost above a threshold. The threshold is smaller when the power spectral density of the noise is biased towards the frequencies that are able to destabilize the system. Recalling figure 6B, the band of frequencies that are able to destabilize the cluster synchronized solution is between 0.008 and 0.04. As a result, pink noise, that has higher low band power spectrum–-11dB $= 0.28$ at frequency 0.01– destabilizes the cluster synchronized solution with noise intensity $\varepsilon \sim 1.5$ (note that $1.5 \times 0.28 = 0.42$, that is approximately the threshold we have found in Supplementary Figure 6A). White noise has a lower low band power spectrum –-17dB $= 0.14$ at frequency 0.01–and requires a larger noise intensity $\varepsilon \sim 2.8$ (note that $2.8 \times 0.14 = 0.39$) to yield DLS desynchronization. Finally, blue noise, the one most biased on the higher frequencies –-22dB $= 0.08$ at frequency 0.01– is the one for which cluster synchronization is more robust, requiring intensity $\varepsilon \sim 4.8$ (note that $4.8 \times 0.08 = 0.38$) to destabilize the DLS CS solution.

Supplementary Note 5: Effects of the intra- and inter-layer connectivity strengths on CS stability

Here we consider a situation for which the structure of the inter-layer connections does not affect the group of symmetry of the multilayer network. This is the case in which the compatibility relations $gA^{\alpha\beta} = A^{\alpha\beta}h$ and $hA^{\beta\alpha} = A^{\beta\alpha}g$ are satisfied for all the possible symmetries in the different layers, *e.g.,* the case with no inter-layer connections ($A^{\alpha\beta} = (A^{\beta\alpha})^T = \mathbf{0}_{N^\alpha \times N^\beta}$, $\forall\alpha,\beta$) or all-to-all inter-layer connections ($A^{\alpha\beta} = (A^{\beta\alpha})^T = \mathbf{1}_{N^\alpha \times N^\beta}$, $\forall\alpha,\beta$) –note that other non-trivial cases are possible. Then, $\mathcal{K}_1^\alpha = \mathcal{G}_1^\alpha$, $\forall\alpha$, and as a result all the symmetries of each layer are ILS-symmetries of the multilayer network.

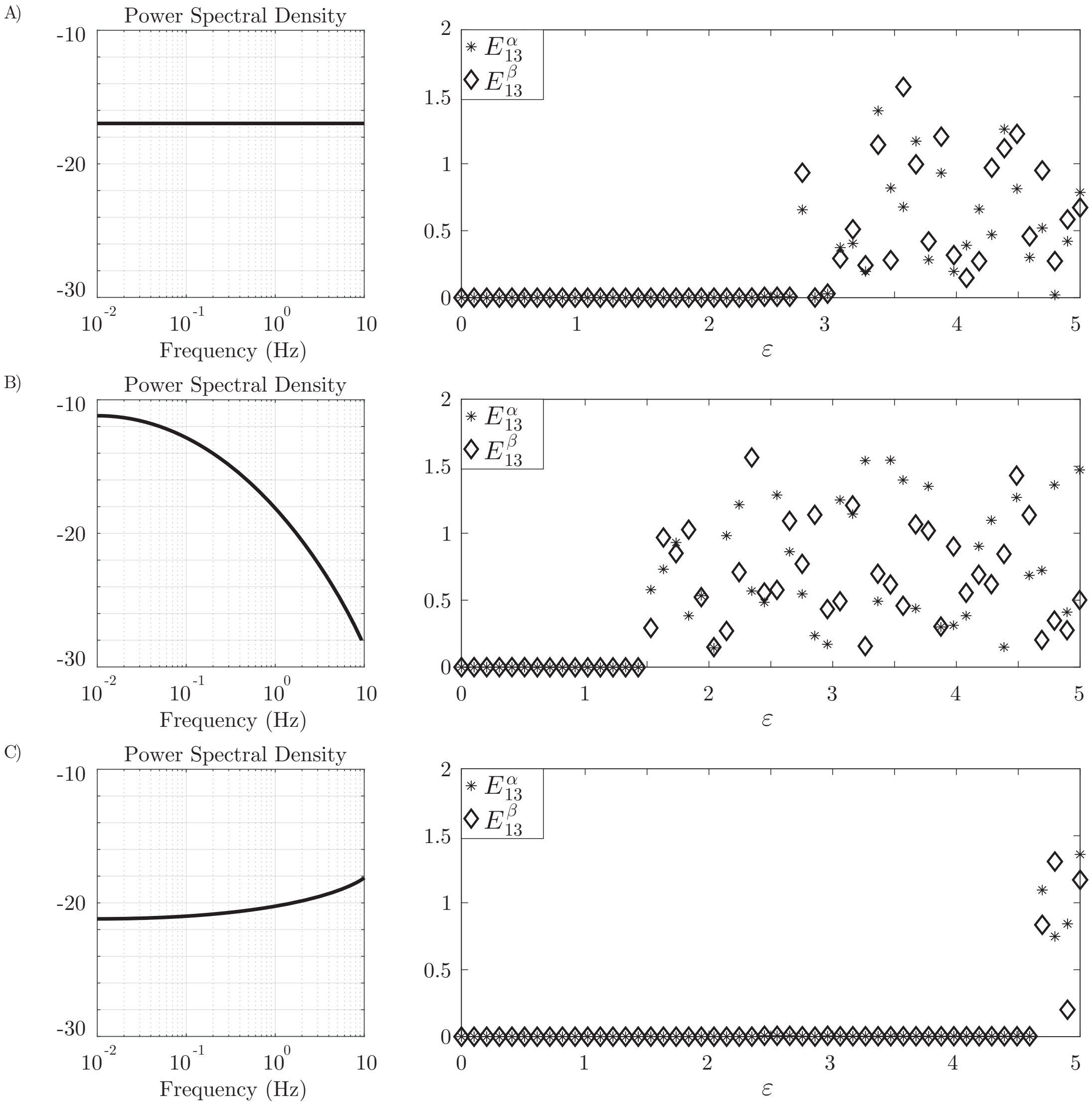

Supplementary Figure 7: Synchronization errors E_{13}^{α} (asterisks) and E_{13}^{β} (diamonds) vs. noise intensity ε when the noise is A) white, B) pink, and C) blue. See text for more details

Consider for example the multilayer network in Figure 1 of the main manuscript, but with all-to-all inter-layer connections. Moreover, let us consider different inter-layer coupling strengths $\sigma^{\alpha\beta}$. While the matrix T remains the same one reported in Eq. 28 of the main manuscript, the matrix $B = TAT^T$ becomes:

$$B = \left[\begin{array}{cccc|cccc} 2\sigma^{\alpha\alpha} & 2\sigma^{\alpha\alpha} & 2\sqrt{2}\sigma^{\alpha\beta} & 2\sigma^{\alpha\beta} & 0 & 0 & 0 & 0 \\ 2\sigma^{\alpha\alpha} & 0 & \sqrt{2}\sigma^{\alpha\beta} & \sigma^{\alpha\beta} & 0 & 0 & 0 & 0 \\ 2\sqrt{2}\sigma^{\alpha\beta} & \sqrt{2}\sigma^{\alpha\beta} & 0 & \sqrt{2}\sigma^{\beta\beta} & 0 & 0 & 0 & 0 \\ 2\sigma^{\alpha\beta} & \sigma^{\alpha\beta} & \sqrt{2}\sigma^{\beta\beta} & 0 & 0 & 0 & 0 & 0 \\ \hline 0 & 0 & 0 & 0 & -2\sigma^{\alpha\alpha} & 0 & 0 & 0 \\ 0 & 0 & 0 & 0 & 0 & 0 & 0 & 0 \\ 0 & 0 & 0 & 0 & 0 & 0 & 0 & 0 \\ 0 & 0 & 0 & 0 & 0 & 0 & 0 & 0 \end{array}\right] \begin{array}{l} \left.\vphantom{\begin{array}{c}a\\a\\a\\a\end{array}}\right\} \text{SYNC block} \\ \left.\vphantom{\begin{array}{c}a\\a\\a\\a\end{array}}\right\} \text{ILS blocks} \end{array} \tag{11}$$

Note that the transverse blocks are, as expected, all ILS blocks, since the intra-layer coupling is all-to-all. It is therefore possible to identify a maximum transversal Lyapunov Exponent for each of the layers. Moreover, in this specific case, all the transverse blocks are one-dimensional. This is a consequence of the particular networks considered on each layer: in fact, without considering the inter-layer couplings, each layer has no intertwining symmetry breakings. We now want to answer the following question: under this setup, how is cluster synchronization affected by the intra- and inter-layer coupling strength? To answer this question, we focus our attention on the case of low coupling (reference value for $\sigma^{\alpha\alpha}$, $\sigma^{\alpha\beta}$, $\sigma^{\beta\beta}$ is 0.01), and we take, as an example, the Van der Pol model discussed in the previous section, with $\delta_c = 0.5$.

Supplementary Figure 8 shows that if we increase only the intra-layer coupling $\sigma^{\alpha\alpha}$, we can achieve cluster-synchronization in the layer α; on the other hand, the cluster-synchronized solution of layer β becomes unstable as $\sigma^{\alpha\alpha}$ increases. If instead we leave the intra-layer coupling strength unvaried and we increase only the inter-layer coupling strength $\sigma^{\alpha\beta}$, we see that cluster synchronization is achieved in both the layers. This simple example highlights the importance of properly tuning the inter-layer coupling strengths, which is key in achieving cluster synchronization in both the layers.

Supplementary Note 6: Stability analysis of the three CS patterns possible in the experiment

From the analysis of the group of symmetries of the multilayer network, we have identified three possible clustered synchronized patterns. To analyze the stability of each pattern we write down a suitable T matrix.

Pattern 1a We now write down the T^α, T^β and T^γ matrices of the multilayer network corresponding to pattern 1a. Layer α includes only one node, layer β and γ are fully synchronized, thus:

$$T^\alpha_{1a} = [1], \quad T^\beta_{1a} = \begin{bmatrix} \frac{1}{\sqrt{2}} & \frac{1}{\sqrt{2}} \\ \frac{1}{\sqrt{2}} & -\frac{1}{\sqrt{2}} \end{bmatrix}, \quad T^\gamma_{1a} = \begin{bmatrix} \frac{1}{2} & \frac{1}{2} & \frac{1}{2} & \frac{1}{2} \\ \frac{1}{2} & \frac{1}{2} & -\frac{1}{2} & -\frac{1}{2} \\ \frac{1}{\sqrt{2}} & -\frac{1}{\sqrt{2}} & 0 & 0 \\ 0 & 0 & \frac{1}{\sqrt{2}} & -\frac{1}{\sqrt{2}} \end{bmatrix}.$$

The first row of each T matrix describes the synchronous solution, while the remaining rows describe motion transverse to the synchronous manifold. The matrix T that we apply to transform the system

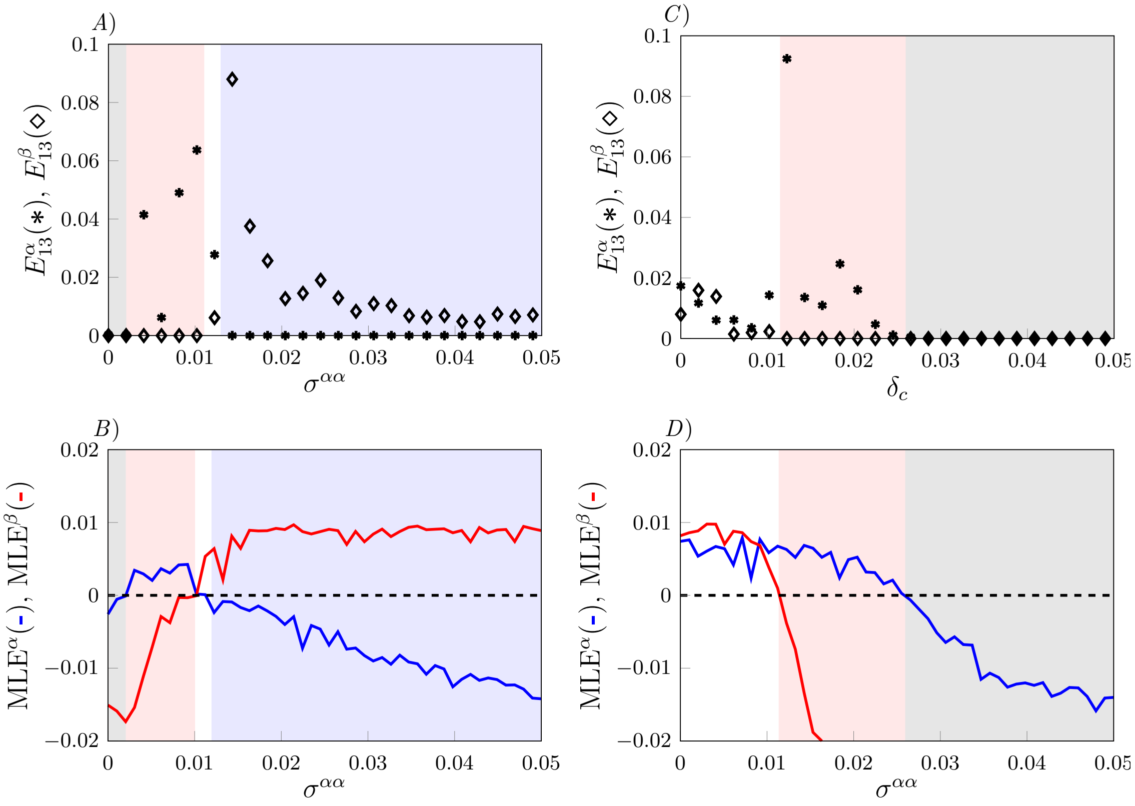

Supplementary Figure 8: Synchronization errors E_{13}^{α} (asterisks) and E_{13}^{β} (diamonds) (top panels) and transversal MLEs (bottom panels) vs the intra-layer coupling strength $\sigma^{\alpha\alpha}$ (left) and the inter-layer coupling strength $\sigma^{\alpha\beta}$ (right). Blue/red shadowed area indicates cluster synchronization only on the α/β layer, respectively, while in gray shadowed area both the layers are clustered synchronized. Increasing $\sigma^{\alpha\alpha}$ it is possible to achieve cluster synchronization in layer α (approximately, with $\sigma^{\alpha\alpha} > 0.012$) but we cannot obtain cluster synchronization in layer β. Conversely, increasing the inter-layer coupling strength $\sigma^{\alpha\beta}$, cluster synchronization can be achieved in both the layers.

is the direct sum of the three matrices, *i.e.,*

$$T_{1a} = \begin{bmatrix} 1 & 0 & 0 & 0 & 0 & 0 & 0 \\ 0 & \frac{1}{\sqrt{2}} & \frac{1}{\sqrt{2}} & 0 & 0 & 0 & 0 \\ 0 & 0 & 0 & \frac{1}{2} & \frac{1}{2} & \frac{1}{2} & \frac{1}{2} \\ \hline 0 & \frac{1}{\sqrt{2}} & -\frac{1}{\sqrt{2}} & 0 & 0 & 0 & 0 \\ 0 & 0 & 0 & \frac{1}{2} & \frac{1}{2} & -\frac{1}{2} & -\frac{1}{2} \\ \hline 0 & 0 & 0 & \frac{1}{\sqrt{2}} & -\frac{1}{\sqrt{2}} & 0 & 0 \\ \hline 0 & 0 & 0 & 0 & 0 & \frac{1}{\sqrt{2}} & -\frac{1}{\sqrt{2}} \end{bmatrix} \begin{matrix} \left.\vphantom{\begin{matrix}1\\1\\1\end{matrix}}\right\} \text{1a sync manifold} \\ \left.\vphantom{\begin{matrix}1\\1\end{matrix}}\right\} \text{transverse direction toward 2} \\ \left.\vphantom{\begin{matrix}1\\1\end{matrix}}\right\} \text{transverse directions toward 1b} \end{matrix} \qquad (12)$$

where we have reordered the rows of the matrix in order to group them. Note that we have colored lavender (pink) the rows of the matrix T_{1a} that correspond to layer β (γ.) Applying the transformation to the multilayer network we obtain the transformed coupling matrices

$$B^{\alpha\beta} = \left(B^{\beta\alpha}\right)^T = [\sqrt{2}, 0], \quad B^{\beta\beta} = \begin{bmatrix} 0 & 0 \\ 0 & -2 \end{bmatrix}, \quad B^{\beta\gamma} = \left(B^{\gamma\beta}\right)^T = \begin{bmatrix} \sqrt{2} & 0 & 0 & 0 \\ 0 & \sqrt{2} & 0 & 0 \end{bmatrix}, \quad B^{\gamma\gamma} = \begin{bmatrix} 1 & 0 & 0 & 0 \\ 0 & 1 & 0 & 0 \\ 0 & 0 & -1 & 0 \\ 0 & 0 & 0 & -1 \end{bmatrix},$$

In accordance with the notation introduced in the main manuscript, we write the supra-adjacency matrix A and the matrix B,

$$A = \begin{bmatrix} 0 & A^{\alpha\beta} & 0 \\ A^{\beta\alpha} & A^{\beta\beta} & A^{\beta\gamma} \\ 0 & A^{\gamma\beta} & A^{\gamma\gamma} \end{bmatrix} \qquad B = \begin{bmatrix} 0 & B^{\alpha\beta} & 0 \\ B^{\beta\alpha} & B^{\beta\beta} & B^{\beta\gamma} \\ 0 & B^{\gamma\beta} & B^{\gamma\gamma} \end{bmatrix}.$$

Reordering the rows and columns of B in order to better identify its block structure, we obtain,

$$B = \left[\begin{array}{ccc|cc|c|c} 0 & \sqrt{2} & 0 & 0 & 0 & 0 & 0 \\ \sqrt{2} & 0 & \sqrt{2} & 0 & 0 & 0 & 0 \\ 0 & \sqrt{2} & 1 & 0 & 0 & 0 & 0 \\ \hline 0 & 0 & 0 & -2 & \sqrt{2} & 0 & 0 \\ 0 & 0 & 0 & \sqrt{2} & 1 & 0 & 0 \\ \hline 0 & 0 & 0 & 0 & 0 & -1 & 0 \\ \hline 0 & 0 & 0 & 0 & 0 & 0 & -1 \end{array}\right] \begin{matrix} \left.\vphantom{\begin{matrix}1\\1\\1\end{matrix}}\right\} \text{LEs of the sync manifold} \\ \left.\vphantom{\begin{matrix}1\\1\end{matrix}}\right\} B^2 \ \} \text{LEs transverse to solutions 1} \\ \left.\vphantom{1}\right\} B_1^{1b} \\ \left.\vphantom{1}\right\} B_2^{1b} \ \} \text{LEs transverse to solutions 1a and 2} \end{matrix} \qquad (13)$$

(the same result can be obtained directly applying the transformation T_{1a} to the matrix A). The fully synchronous solution has 3 transverse blocks. The first transverse block B^2 corresponds to the breaking of a DLS and is associated with a perturbation of the form of Eq. 27 of the main manuscript; this describes the breaking of the β layer, which also splits the γ layer into two clusters. This gives solutions of kind 2. There are a total of $N^\beta + N^\gamma = 5$ LEs associated with this block.

The second and the third transverse blocks B_1^{1b} and B_2^{1b} (each composed of $N^\gamma = 3$ LEs) correspond to ILS breakings and are associated with perturbations of the form of Eq. 26 of the main manuscript. They describe the splitting of the γ layer into two clusters, giving solutions of kind 1b. Note that the two blocks are equal: this means both breakings have the same stability property. This is because the two breakings describe the same kind of behavior; the only difference between the two breakings is the labeling order used in the γ layer.

The quotient network 1a is always monostable. In Supplementary Figure 9(a), we show the MLEs of B^2 (solid) and B^{1b} (dashed). This figure is the same as Figure 5 of the main manuscript and is copied here for the reader's convenience. Note that the 1a solution is stable when both the MLEs are negative, i.e. only if $k_C > 0.2$.

The synchronous manifold 1a is contained in both the synchronous manifold 1b and the synchronous manifold 2. Thus, this diagram also shows the region where the 1a solution is present and stable in the other two manifolds. The symmetry breaking (pitchfork) bifurcations where each of the two curves change sign are highlighted in the figure with vertical lines, red if the breaking involves a solution of type 1b, yellow if the breaking involves a solution of type 2.

Patterns 1b

Two equivalent patterns of type 1b are possible, due to the different possible labelings in the γ layer (i.e., Colpitts 1 & 2 and 3 & 4 are interchangeable in the network). These patterns can be obtained applying two last breakings of T^γ_{1a} (labeled as 'transverse directions towards 1b' in Eq. (12)) to the synchronous manifold. The two possible 1b patterns have therefore:

$$T^\alpha_{1b} = T^\alpha_{1a}, \quad T^\beta_{1b} = T^\beta_{1a}, \quad T^\gamma_{1b,1} = \begin{bmatrix} \frac{1}{\sqrt{2}} & 0 & \frac{1}{\sqrt{2}} & 0 \\ 0 & \frac{1}{\sqrt{2}} & 0 & \frac{1}{\sqrt{2}} \\ \frac{1}{\sqrt{2}} & 0 & -\frac{1}{\sqrt{2}} & 0 \\ 0 & \frac{1}{\sqrt{2}} & 0 & -\frac{1}{\sqrt{2}} \end{bmatrix}, \text{ or } T^\gamma_{1b,2} = \begin{bmatrix} \frac{1}{\sqrt{2}} & 0 & 0 & \frac{1}{\sqrt{2}} \\ 0 & \frac{1}{\sqrt{2}} & \frac{1}{\sqrt{2}} & 0 \\ \frac{1}{\sqrt{2}} & 0 & 0 & -\frac{1}{\sqrt{2}} \\ 0 & \frac{1}{\sqrt{2}} & -\frac{1}{\sqrt{2}} & 0 \end{bmatrix}$$

The resulting T^{1b} and B matrices are:

$$T_{1b,1} = \left[\begin{array}{ccccccc} 1 & 0 & 0 & 0 & 0 & 0 & 0 \\ 0 & \frac{1}{\sqrt{2}} & \frac{1}{\sqrt{2}} & 0 & 0 & 0 & 0 \\ 0 & 0 & 0 & \frac{1}{\sqrt{2}} & 0 & 0 & \frac{1}{\sqrt{2}} \\ 0 & 0 & 0 & 0 & \frac{1}{\sqrt{2}} & \frac{1}{\sqrt{2}} & 0 \\ \hline 0 & \frac{1}{\sqrt{2}} & -\frac{1}{\sqrt{2}} & 0 & 0 & 0 & 0 \\ 0 & 0 & 0 & \frac{1}{\sqrt{2}} & 0 & 0 & -\frac{1}{\sqrt{2}} \\ 0 & 0 & 0 & 0 & \frac{1}{\sqrt{2}} & -\frac{1}{\sqrt{2}} & 0 \end{array}\right], \quad B = \left[\begin{array}{cccc|ccc} 0 & \sqrt{2} & 0 & 0 & 0 & 0 & 0 \\ \sqrt{2} & 0 & 1 & 1 & 0 & 0 & 0 \\ 0 & 1 & 0 & 1 & 0 & 0 & 0 \\ 0 & 1 & 1 & 0 & 0 & 0 & 0 \\ \hline 0 & 0 & 0 & 0 & -2 & 1 & 1 \\ 0 & 0 & 0 & 0 & 1 & 0 & 1 \\ 0 & 0 & 0 & 0 & 1 & 1 & 0 \end{array}\right].$$

$$T_{1b,2} = \left[\begin{array}{ccccccc} 1 & 0 & 0 & 0 & 0 & 0 & 0 \\ 0 & \frac{1}{\sqrt{2}} & \frac{1}{\sqrt{2}} & 0 & 0 & 0 & 0 \\ 0 & 0 & 0 & \frac{1}{\sqrt{2}} & 0 & \frac{1}{\sqrt{2}} & 0 \\ 0 & 0 & 0 & 0 & \frac{1}{\sqrt{2}} & 0 & \frac{1}{\sqrt{2}} \\ \hline 0 & \frac{1}{\sqrt{2}} & -\frac{1}{\sqrt{2}} & 0 & 0 & 0 & 0 \\ 0 & 0 & 0 & \frac{1}{\sqrt{2}} & 0 & -\frac{1}{\sqrt{2}} & 0 \\ 0 & 0 & 0 & 0 & \frac{1}{\sqrt{2}} & 0 & -\frac{1}{\sqrt{2}} \end{array}\right], \quad B = \left[\begin{array}{cccc|ccc} 0 & \sqrt{2} & 0 & 0 & 0 & 0 & 0 \\ \sqrt{2} & 0 & 1 & 1 & 0 & 0 & 0 \\ 0 & 1 & 0 & 1 & 0 & 0 & 0 \\ 0 & 1 & 1 & 0 & 0 & 0 & 0 \\ \hline 0 & 0 & 0 & 0 & -2 & 1 & 1 \\ 0 & 0 & 0 & 0 & 1 & 0 & 1 \\ 0 & 0 & 0 & 0 & 1 & 1 & 0 \end{array}\right].$$

As expected, the two B matrices are equal (in fact, the stability property of each of these solutions are the same). The 3-dimensional transverse block of the 1b clustered solution corresponds to the breaking of a DLS and is associated with a perturbation of the form of Eq. 27 of the main manuscript and it indicates that it is not possible for the γ layer to desynchronize while the β layer is synchronized.

The transverse block is composed by $N^\beta + 2N^\gamma = 8$ LEs; it describes the breaking of the β and γ layers, giving rise to solutions without any clustered synchronization.

Solution 1a is also present when we reside on the 1b synchronous manifold, see Figure 9(b). Since no catastrophic bifurcation destroys this solution, it is always present; it is stable for the k_C values for which the dotted curve in figure 9(a) is negative, and unstable otherwise. When the dotted curve of figure 9(a) crosses zero, a symmetry-breaking bifurcation (pitchfork bifurcation) occurs on the 1b invariant manifold. The first crossing from the right, at $k_c \approx 0.08$, is a supercritical pitchfork bifurcation: the solution of type 1a loses its stability and another solution of type 1b homotopically emerges. This solution exists for all $k_c < 0.08$. The 1a solution regains stability at $k_C = 0$; in this case the pitchfork bifurcation is subcritical. This means that the 1a solution becomes stable because an unstable solution of type 1b is destroyed at that point. That unstable solution was generated at an analogous bifurcation at $k_C = -0.22$, where, again, the type 1a solution loses stability. In the region $k_c \in [-0.22, 0]$ the quotient system 1b is bistable, with a solution of type 1a and a solution of type 1b; it is otherwise monostable.

The maximum transverse Lyapunov exponent computed around each solution is shown in Supplementary Figure 9(b), in blue when the solution is of type 1a, in red when the solution is of type 1b.

Pattern 2

The resulting block structure in (13) shows that the breaking of the β layer (second row of the matrix T^{β}_{1a}) and the first breaking of the γ layer (second row of the matrix T^{γ}_{1a}) intertwine. Therefore, they must be applied together in order to break pattern 1a towards pattern 2 (as labeled in (12)). So doing, we obtain the following transformations for each layer:

$$T_2^{\alpha} = T_{1a}^{\alpha}, \quad T_2^{\beta} = \begin{bmatrix} 1 & 0 \\ 0 & 1 \end{bmatrix}, \quad T_2^{\gamma} = \begin{bmatrix} \frac{1}{\sqrt{2}} & \frac{1}{\sqrt{2}} & 0 & 0 \\ 0 & 0 & \frac{1}{\sqrt{2}} & \frac{1}{\sqrt{2}} \\ \frac{1}{\sqrt{2}} & -\frac{1}{\sqrt{2}} & 0 & 0 \\ 0 & 0 & \frac{1}{\sqrt{2}} & -\frac{1}{\sqrt{2}} \end{bmatrix}.$$

The resulting T_2 and B matrices are:

$$T_2 = \begin{bmatrix} 1 & 0 & 0 & 0 & 0 & 0 & 0 \\ 0 & 1 & 0 & 0 & 0 & 0 & 0 \\ 0 & 0 & 1 & 0 & 0 & 0 & 0 \\ 0 & 0 & 0 & \frac{1}{\sqrt{2}} & \frac{1}{\sqrt{2}} & 0 & 0 \\ 0 & 0 & 0 & 0 & 0 & \frac{1}{\sqrt{2}} & \frac{1}{\sqrt{2}} \\ \hline 0 & 0 & 0 & \frac{1}{\sqrt{2}} & -\frac{1}{\sqrt{2}} & 0 & 0 \\ \hline 0 & 0 & 0 & 0 & 0 & \frac{1}{\sqrt{2}} & -\frac{1}{\sqrt{2}} \end{bmatrix}, \quad B = \left[\begin{array}{ccccc|c|c} 0 & 1 & 1 & 0 & 0 & 0 & 0 \\ 1 & -1 & 1 & \sqrt{2} & 0 & 0 & 0 \\ 1 & 1 & -1 & 0 & \sqrt{2} & 0 & 0 \\ 0 & \sqrt{2} & 0 & 1 & 0 & 0 & 0 \\ 0 & 0 & \sqrt{2} & 0 & 1 & 0 & 0 \\ \hline 0 & 0 & 0 & 0 & 0 & -1 & 0 \\ \hline 0 & 0 & 0 & 0 & 0 & 0 & -1 \end{array}\right].$$

The matrix B has two identical transverse blocks corresponding to ILS breakings and are associated with perturbations of the form of Eq. 26 of the main manuscript. These two blocks can be studied separately, indicating that the separation of Colpitts 1 from 2 and of Colpitts 3 from 4 are not interdependent (as we expect, since they do not communicate). However, since the LEs are governed by the same equation, we expect when one pair breaks the other should also break (they are identical, and there is no reason why two Colpitts should maintain their synchrony while the other two should not).

As stated earlier when discussing stability of pattern (1a), if we reside on the 2 synchronous manifold, the 1a solution is present when the solid line in Supplementary Figure 9(a) is negative. Like the 1b manifold, no catastrophic bifurcations destroy the solution of type 1a; it is always

present, either stable or unstable, see Supplementary Figure 9(c). Both the stability changes are due to supercritical pitchfork bifurcations, involving a stable symmetry-broken solution of type 2. The leftmost supercritical pitchfork bifurcation, at $k_C = -0.32$, generates a solution that exists up to $k_C = 0.14$, where it undergoes a saddle-node bifurcation and it is destroyed. The solution that is generated at the supercritical pitchfork bifurcation at $k_C = 0.2$ is also destroyed through a saddle-node bifurcation at $k_C = 0$. This manifold, thus, supports bistability when $k_c \in (0, 0.14)$.

Around each stable solution on the manifold, we compute the two groups of N^γ (identical) transverse LEs; figure 9(c) shows the maximum LE values corresponding to solution 1a (blue) and solution 2 (yellow).

Supplementary Note 7: Experimental observations

Different clustered patterns are identified in the experiment by a combined analysis of the phases and frequencies of the individual experimental oscillators.

Supplementary Figure 10 shows the observed cluster states of the experimental system as inductive Colpitts coupling k_c is varied. In the top wide panels, we show four phase differences as k_c is varied: $\Delta\phi_{F12} = \phi_{\mathrm{FHN}_2} - \phi_{\mathrm{FHN}_1}$; $\Delta\phi_{C12} = \phi_{\mathrm{Colp}_2} - \phi_{\mathrm{Colp}_1}$; $\Delta\phi_{C13} = \phi_{\mathrm{Colp}_3} - \phi_{\mathrm{Colp}_1}$; and $\Delta\phi_{C34} = \phi_{\mathrm{Colp}_4} - \phi_{\mathrm{Colp}_3}$.

Again, phase is calculated from the peaks of the oscillations and linearly interpolated in between. The average phase difference which is plotted in the top panels of Supplementary Figure 10 is calculated over 0.12 ms. Note that when the phase difference is not stationary, this value is not truly representative of the dynamics; however the average phase difference is a useful way of visually differentiating the different clustered behaviors.

In the top wide panel, experiments occur from right to left. We begin on the right side of the top panel with large positive coupling (when the inductors are close and anti-parallel); we travel leftward on this figure as k_c is reduced (by increasing the separation of the inductors). We cross $k_c = 0$ by switching the inductors from anti-parallel to parallel, and then we bring the inductors closer to reach maximum parallel inductive coupling with $k_c = -0.4$. We reverse this procedure to produce the bottom wide panel, in which experiments occur from left to right.

Supplementary Figure 11 shows the frequencies of the oscillators in the cluster states shown in Supplementary Figure 10. The oscillator frequencies are an additional tool for differentiating between cluster states; we see that different cluster states have different frequencies and changes in the magnetic coupling produce different changes depending upon the cluster state.
Comments on Supplementary Figure 10 and Supplementary Figure 11:

- Pattern 1a (green in Supplementary Figures 10 and 11) exists with strong anti-parallel coupling (k_C large and positive). Pattern 1a is globally stable for a small range of k_c; it is bistable with pattern 2-($\pi/2$) for another range of k_c. All oscillators have the same frequency; the frequency increases as k_c decreases.

- Pattern 2 exists with two configurations: pattern 2-($\pi/2$) (yellow in Supplementary Figures 10 and 11) has a lower frequency and a phase offset around $\pi/2$; pattern 2-(π) (lavender in Supplementary Figures 10 and 11) has a higher frequency and a phase offset around π. Pattern 2-($\pi/2$) has two regions of bistability (with pattern 2-(π) and pattern 1a) and one region of monostability. Pattern 2-(π) has two additional regions of bistability (with pattern 1b and the no pattern) and its own region of monostability.

- Pattern 1b (blue in Supplementary Figures 10 and 11) exists with strong parallel inductive coupling. Here, $\Delta\phi_{C12} = \Delta\phi_{C43} = \pi$. We expect $\Delta\phi_{C13} = \pi$ or 0, since there are two possible indistinguishable patterns 1b; this is true for some experiments in this region. For others, the Colpitts pairs 1/2 and 3/4 oscillate at slightly different frequencies, which causes the phase difference between the left and right Colpitts $\Delta\phi_{C13}$ (red curve on the figure) to slowly slip. As we stated, this averaged phase difference does not represent a stationary behavior, and merely serves to visually distinguish the clustered state 1b from the modified version we identify in the theory section in which synchronization is not achieved (positive but small MLE, red curve in Figure 6 of the main manuscript). This result is in accordance with the theoretical prediction: in this region the MLE associated with solution 1b varies around zero, undergoing many supercritical symmetry breaking bifurcations. In Supplementary Figure 11, we can see the slight frequency differences between the Colpitts pairs 1 & 2 and 3 & 4; the Colpitts oscillate considerably faster than the FHNs.

- No pattern (white in Supplementary Figures 10 and 11) exists with weak parallel inductive coupling. Again, the oscillators are not frequency locked. Again, the averaged phase difference shown in figure 10 does not have a real meaning, and serves to identify the white shaded region. This state appears to be related to the pattern 2-($\pi/2$) solution, as the phase difference $\Delta\phi_{F12}$ slows down near (but passes through) $\Delta\phi_{F12} = \pi/2$.

Supplementary Figure 11 shows the frequencies of the oscillators in the cluster states shown in Supplementary Figure 10; again, the upper panel reads from right to left, followed by the lower panel which reads from left to right. The shading again refers to the cluster state. The oscillator frequencies are an additional tool for differentiating between cluster states; we see that different cluster states have different frequencies and changes in the magnetic coupling produce different changes depending upon the cluster state. We show the frequencies of both FHNs and all four Colpitts oscillators.

Supplementary Figure 12 shows the phase differences of the observable cluster states reported in Figure 6 of the main manuscript This figure bridges the theoretical predictions and the experimental cluster observations which we identify by both phase difference and frequency. Phase is calculated from the peaks of the oscillations and linearly interpolated in between. We show three phase differences: $\Delta\phi_{F12} = \phi_{\mathrm{FHN}_2} - \phi_{\mathrm{FHN}_1}$ (black); $\Delta\phi_{C12} = \phi_{\mathrm{Colp}_2} - \phi_{\mathrm{Colp}_1}$ (blue); and $\Delta\phi_{C13} = \phi_{\mathrm{Colp}_3} - \phi_{\mathrm{Colp}_1}$ (red). We do not report the fourth phase difference, $\phi_{\mathrm{Colp}_3} - \phi_{\mathrm{Colp}_4}$; because Colpitts 1 & 2 and 3 & 4 are interchangeable, this phase difference is identical to $\Delta\phi_{C12}$. We repeat the shading from Figure 6 of the main manuscript. In the bottom panel, we show the Pattern 2-(π) solution; in the top panel we show all other solutions. We show no phase difference in the top panel for $0 < k_C < 0.07$; in this region we see an unsynchronous solution and thus there is no average phase difference. We can see the slightly desynchronized pattern 1b solutions that occur in the blue region when the red curve from Figure 6 of the main manuscript is positive; in these regions the phase difference $\Delta\phi_{C12}$ varies slightly from π and the phase differences $\Delta\phi_{F12}$ and $\Delta\phi_{C13}$ vary slightly from 0.

Supplementary Note 8: Discrepancies between simulation and experiment

The main discrepancy we highlight between simulation and experiment is in the modeling of the FHN circuits. Note that in Figure 3 of the main manuscript, each FHN has four resistances, $R_{1,i}$, $R_{2,i}$, $R_{3,i}$ and $R_{4,i}$, $i = 1, 2$. In the model, only two resistances are present ($R_{1,i}$ and $R_{3,i}$), while only the ratio of the other two appears in the model (see Equation (62) in the paper). Nevertheless, changing the value of these resistances changes the experimental waveform, which is not captured by our modeling framework. As shown in Supplementary Figure 13, changing the value of $R_{2,i} = R_{4,i}$ changes the amplitude, frequency, and waveform of the oscillation; when $R_{2,i} = R_{4,i}$ is sufficiently large, no oscillation occurs.

The model for the transistor 2N2222 of each Colpitts circuit is also simplified; represented with a linear piecewise function. The AD844 operational amplifier is also represented as an ideal component. Finally, component heterogeneity, battery degradation, stray inductance, and experimental noise can also generate differences between the simulations and the experiments. As a result, while we find a good qualitative agreement between the experiment and the simulations, some discrepancies exist. Some of these discrepancies are quantitative:

- theoretically, we expect the blue 2 pattern to survive up to $k_C = -0.27$ while we see this pattern only up to $k_C = -0.16$;
- theoretically, we expect the 1b pattern to survive for all the negative k_C, while experimentally it disappears for $k_C > -0.12$;
- theoretically, we expect pattern 2-($\pi/2$) to survive down to $k_C = 0.07$, while experimentally it disappears for $k_C < 0.12$.

In these cases, we lose a solution experimentally before its theoretical loss of stability. The center and stable manifolds of the unstable (saddle) solutions delimit the basin of attraction of the stable solution. When we approach the bifurcation, the basin of attraction of the stable solution shrinks; the unstable limit cycles collapse around the stable limit cycle, and the solution must stay strictly in this shrinking region to retain the solution it represents. If the experimental noise knocks the trajectory out of this region, it can cause the solution to transition earlier than we would expect in the absence of noise.

Two other qualitative discrepancies are present:

- A region of bistability between green and yellow exists in experiments, but not in the theory. This can be explained theoretically by the heterogeneity of the system; in fact, simulations with heterogeneous components show the symmetry breaking (pitchfork) bifurcation breaks into a limit-point bifurcation, thus generating bistable regions.
- Because the unclustered white region is next to the blue region and not next to the yellow region, one may wonder if the experimental white region is related with some loss of stability of the blue pattern (rather than the yellow one). Analysis of the experimental phase difference of the FHNs, $\Delta\phi_{F12}$, in the white region reveals a preferred phase difference near $\pi/2$; $\Delta\phi_{F12} = \pi/2$ characterizes the yellow pattern (while $\Delta\phi_{F12} = 0$ characterizes the blue pattern). We infer this phase preference because $\Delta\phi_{F12}$ slows down as it passes through $\Delta\phi_{F12} = \pi/2$. This relates the white experimental pattern with the theoretical one. We explain its shift to $k_C < 0$ by the huge role heterogeneity plays when the coupling is small.

The interplay of theory and experiments helped us to understand the system's properties. The FHN and Colpitts oscillators have fundamentally different governing equations and behavior. They are characterized by different levels of noise, and different sources of nonlinearity. As theory seeks to explain natural systems with heterogeneous components, further validations will need to be performed in order to understand where modeling excels and where it can improve.

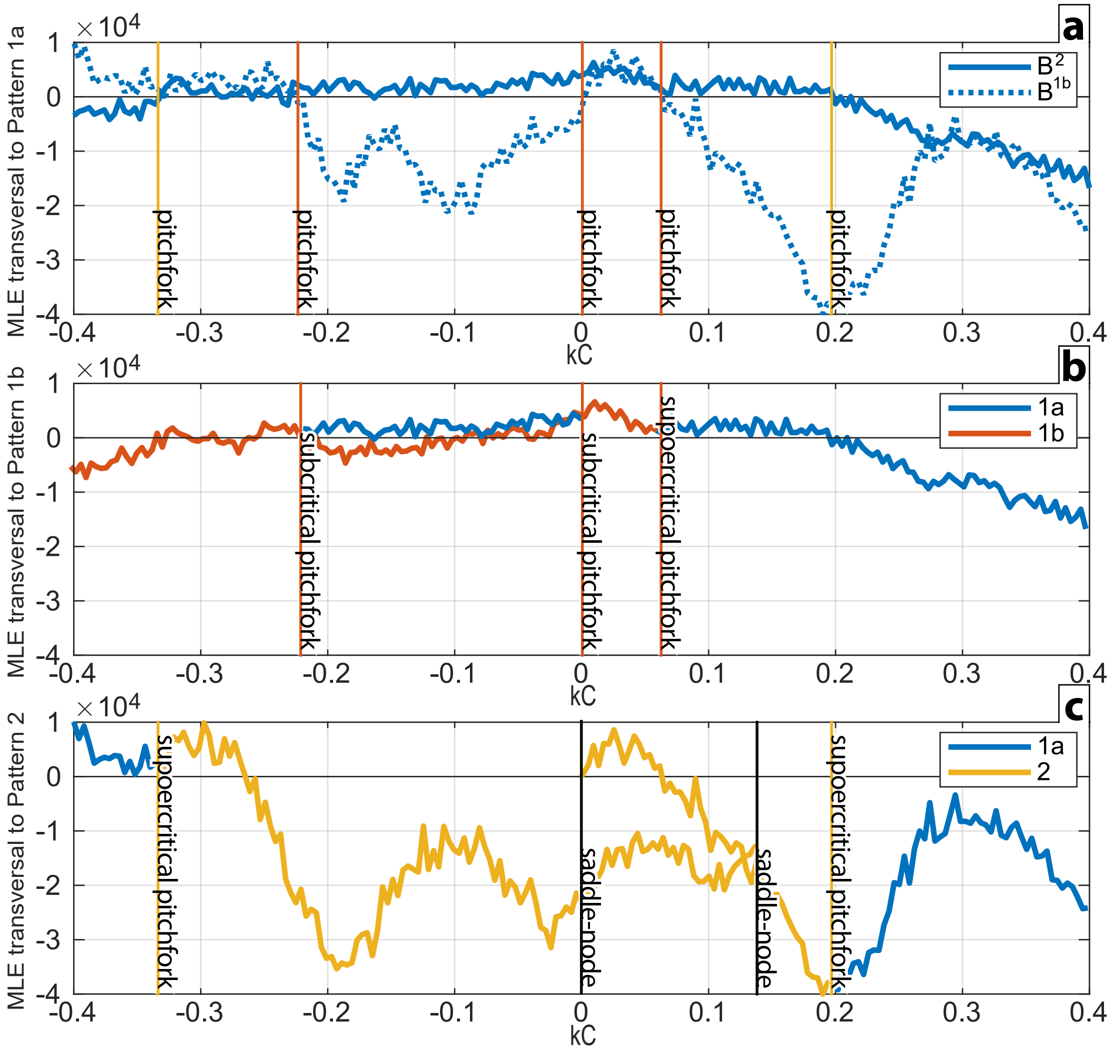

Supplementary Figure 9: **Stability of different cluster synchronization patterns.** (a) MLEs transverse to solution 1a. Solid and dotted lines refer to different transverse blocks. When a line crosses zero it identifies a symmetry breaking bifurcation in one of the other invariant manifold (red: bifurcations on CS manifold 1b - yellow: bifurcations on CS manifold 2). Pattern 1a is stable when both the curves are negative. (b) MLEs transverse to stable solutions on the synchronized manifold 1b. Blue curves refer to the transverse MLE of solution of kind 1a, while the red curve refers to the transverse MLE of solution of kind 1b. Vertical lines indicate the bifurcations of the quotient system. (c) MLEs transverse to stable solutions on the synchronized manifold 2. Blue curves refer to the transverse MLE of solution of kind 1a, while the yellow curves refer to the transverse MLE of solution of kind 2. Vertical lines indicate the bifurcations of the quotient system. Colored lines represent symmetry breaking bifurcation (inferred from (a)), while black lines are other bifurcations.

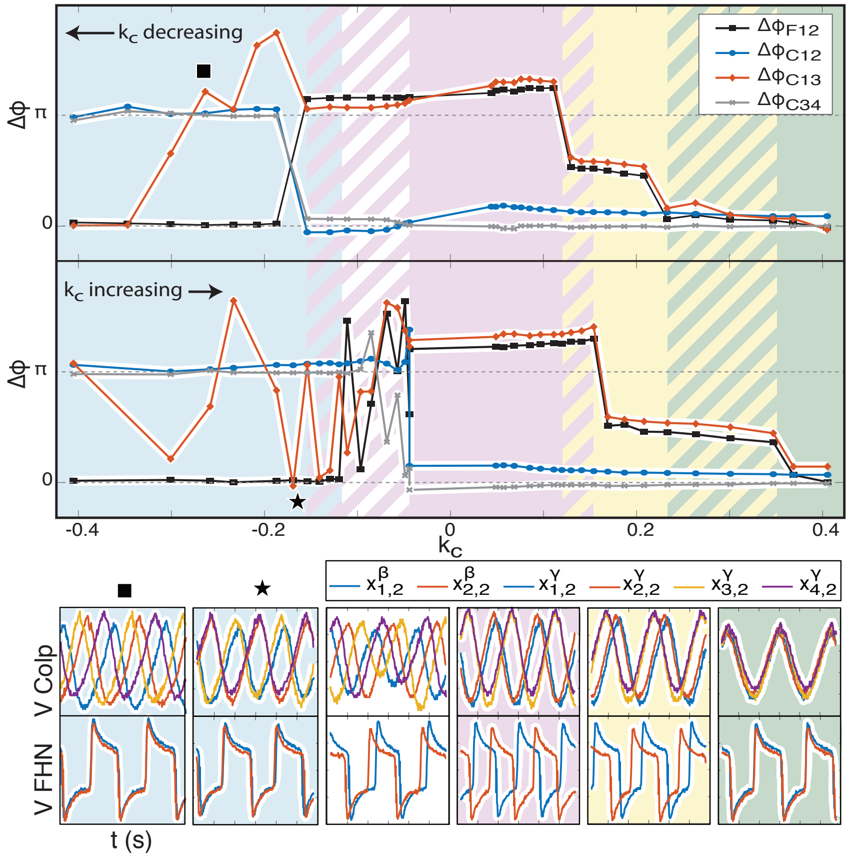

Supplementary Figure 10: **Top:** Cluster configuration as visualized by four phase differences: (black square) $\Delta\phi_{F12} = \phi_{\text{FHN}_2} - \phi_{\text{FHN}_1}$; (blue circle) $\Delta\phi_{C12} = \phi_{\text{Colp}_2} - \phi_{\text{Colp}_1}$; (red diamond) $\Delta\phi_{C13} = \phi_{\text{Colp}_3} - \phi_{\text{Colp}_1}$; (gray x) $\Delta\phi_{C34} = \phi_{\text{Colp}_4} - \phi_{\text{Colp}_3}$. In the top wide panel, we start from the right and reduce inductive coupling between the Colpitts with k_c; in the bottom wide panel, we continue from the left and increase k_c; all experiments were conducted in a single sequence. We observe five kinds of clustered behavior, which are denoted by the shading of the background. Shading with two-colored striping indicates bistability between the two states represented by each color. The frequencies of the oscillators in these experiments is reported in Supplementary Figure 11. **Bottom:** Time series examples of each clustered behavior. (Green) Pattern 1a: all Colpitts are synchronized; all FHNs are synchronized. In top wide panels, all phase differences are zero. (Yellow) Pattern 2-($\pi/2$): Colpitts 1 and 2 are synchronized, Colpitts 3 and 4 are synchronized; the FHNs are separated. In top wide panels, $\Delta\phi_{C12} = 0$, $\Delta\phi_{C34} = 0$, $\Delta\phi_{C13} \neq 0$, and $\Delta\phi_{F12} \neq 0$. (Lavender) Pattern 2-(π): Colpitts 1 and 2 are synchronized, Colpitts 3 and 4 are synchronized; the FHNs are separated. In top wide panels, $\Delta\phi_{C12} = 0$, $\Delta\phi_{C34} = 0$, $\Delta\phi_{C13} \approx \pi$, and $\Delta\phi_{F12} \approx \pi$. (Blue) Pattern 1b: Colpitts 1 is synchronized with Colpitts 3 or 4, Colpitts 2 is synchronized with Colpitts 4 or 3; all FHNs are synchronized. In top wide panels, $\Delta\phi_{C12} \approx \pi$, $\Delta\phi_{C34} \approx \pi$, $\Delta\phi_{C13} = \pi$ or 0 (see discussion), and $\Delta\phi_{F12} = 0$. (White) No pattern: the frequencies of the oscillators become heterogeneous (and therefore the phase differences move with time). (Star) An example of stable solution 1b; (Square) An example of the modified state 1b, as observed in the theory.

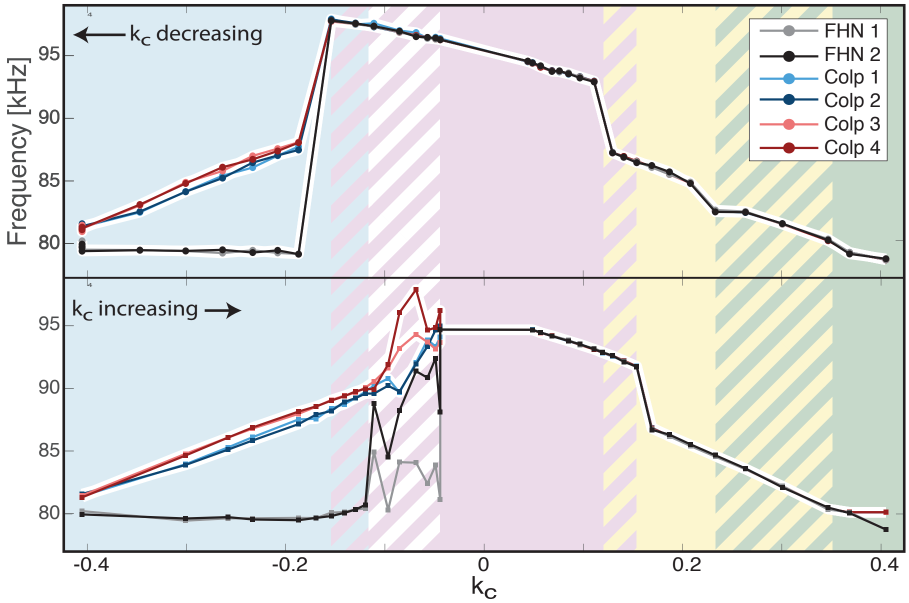

Supplementary Figure 11: Frequencies of the two FHNs (black and gray) and the four Colpitts [C_1 (light blue), C_2 (dark blue), C_3 (light red), C_4 (dark red)] versus the magnetic coupling between the Colpitts pairs, k_c. This figure is computed from the same experimental data as Supplementary Figure 10. In the top panel, we start from the right and reduce inductive coupling between the Colpitts with k_c; in the bottom panel, we continue from the left and increase k_c. The five kinds of clustered behavior are denoted by the shading of the background. Shading with two-colored striping indicates bistability between the two states represented by each color. (Green) Pattern 1a: all Colpitts are synchronized; all FHNs are synchronized. All oscillators have the same frequency, which increases as k_c decreases. (Yellow) Pattern 2-($\pi/2$): Colpitts 1 and 2 are synchronized, Colpitts 3 and 4 are synchronized; the FHNs are separated. All oscillators have the same frequency; Pattern 2-($\pi/2$) is not readily distinguishable from Pattern 1a by frequency. (Lavender) Pattern 2-(π): Colpitts 1 and 2 are synchronized, Colpitts 3 and 4 are synchronized; the FHNs are separated. All oscillators have the same frequency which is substantially faster than the frequency of Pattern 2-($\pi/2$). (Blue) Pattern 1b: Colpitts 1 is synchronized with Colpitts 3 or 4, Colpitts 2 is synchronized with Colpitts 4 or 3; all FHNs are synchronized. The Colpitts are frequency synchronized; the FHNs are frequency synchronized; the two different kinds of oscillators differ substantially in frequency. (White) No pattern: the frequencies of the oscillators become heterogeneous.

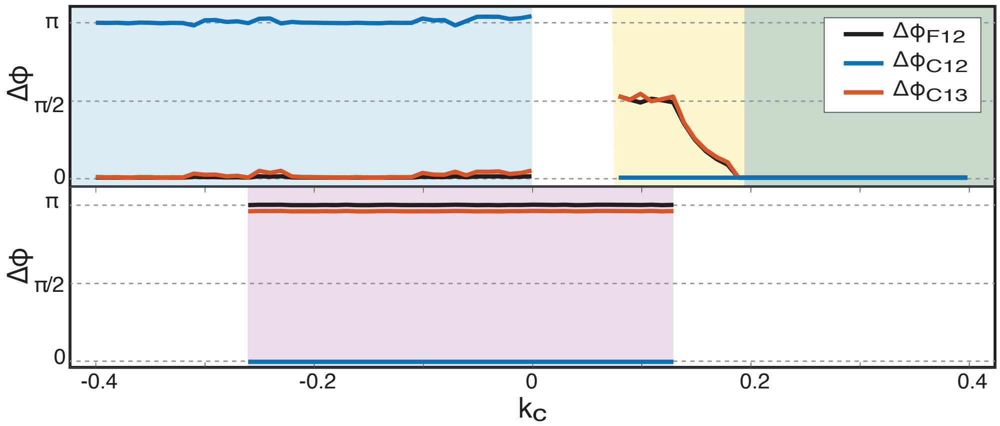

Supplementary Figure 12: Clustered patterns predicted by theoretical analysis as visualized by three phase differences: (black) $\Delta\phi_{F12} = \phi_{\text{FHN}_2} - \phi_{\text{FHN}_1}$; (blue) $\Delta\phi_{C12} = \phi_{\text{Colp}_2} - \phi_{\text{Colp}_1}$; (red) $\Delta\phi_{C13} = \phi_{\text{Colp}_3} - \phi_{\text{Colp}_1}$. **Top:** all clustered solutions other than the pattern 2-(π) solution; **bottom:** the pattern 2-(π) solution. We denote clustered behavior by the shading of the background, as identified and described in Figure 6 of the main manuscript. Shading with two-colored striping indicates bistability between the two states represented by each color. Note that phase difference is shown from 0 to π radians.

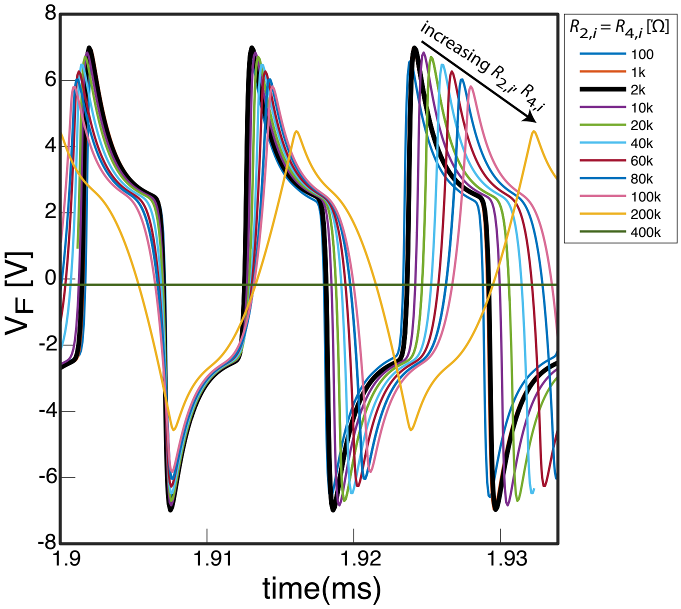

Supplementary Figure 13: Simulation of single FHN using OrCad PSpice software as resistances $R_{2,i} = R_{4,i}$ are increased. Experimental value, $R_{2,i} = R_{4,i} = 2k\Omega$, is shown in black bold.

References

[1] Ryan A. Rossi and Nesreen K. Ahmed. The network data repository with interactive graph analytics and visualization. In *AAAI*, 2015.

[2] Giovanni Filatrella, Arne Hejde Nielsen, and Niels Falsig Pedersen. Analysis of a power grid using a kuramoto-like model. *The European Physical Journal B*, 61(4):485–491, 2008.

[3] Takashi Nishikawa and Adilson E Motter. Comparative analysis of existing models for power-grid synchronization. *New Journal of Physics*, 17(1):015012, 2015.

[4] Benjamin Schäfer, Dirk Witthaut, Marc Timme, and Vito Latora. Dynamically induced cascading failures in power grids. *Nature communications*, 9(1):1–13, 2018.

[5] Jan Machowski, Zbigniew Lubosny, Janusz W Bialek, and James R Bumby. *Power system dynamics: stability and control*. John Wiley & Sons, 2020.

[6] Manlio De Domenico, Albert Solé-Ribalta, Sergio Gómez, and Alex Arenas. Navigability of interconnected networks under random failures. *Proceedings of the National Academy of Sciences*, 111(23):8351–8356, 2014.

[7] Alessio Cardillo, Jesús Gómez-Gardenes, Massimiliano Zanin, Miguel Romance, David Papo, Francisco Del Pozo, and Stefano Boccaletti. Emergence of network features from multiplexity. *Scientific reports*, 3:1344, 2013.

[8] Manlio De Domenico, Andrea Lancichinetti, Alex Arenas, and Martin Rosvall. Identifying modular flows on multilayer networks reveals highly overlapping organization in interconnected systems. *Physical Review X*, 5(1):011027, 2015.

[9] James Coleman, Elihu Katz, and Herbert Menzel. The diffusion of an innovation among physicians. *Sociometry*, 20(4):253–270, 1957.

[10] Manlio De Domenico, Vincenzo Nicosia, Alexandre Arenas, and Vito Latora. Structural reducibility of multilayer networks. *Nature communications*, 6:6864, 2015.

[11] Chris Stark, Bobby-Joe Breitkreutz, Teresa Reguly, Lorrie Boucher, Ashton Breitkreutz, and Mike Tyers. Biogrid: a general repository for interaction datasets. *Nucleic acids research*, 34(suppl_1):D535–D539, 2006.

[12] K-I Goh, Byungnam Kahng, and Doochul Kim. Universal behavior of load distribution in scale-free networks. *Physical review letters*, 87(27):278701, 2001.

[13] Maintainer Gabor Csardi. Package 'igraph'. *Last accessed*, 3(09):2013, 2013.

[14] Isaac Samuel Klickstein and Francesco Sorrentino. Generating graphs with symmetry. *IEEE Transactions on Network Science and Engineering*, 2018.

[15] Isaac Klickstein and Francesco Sorrentino. Generating symmetric graphs. *Chaos: An Interdisciplinary Journal of Nonlinear Science*, 28(12):121102, 2018.
